# Supplementary material for: Climatic niche comparison across a cryptic species complex
Source: PeerJ. 2019 May 31;7:e7042. doi: 10.7717/peerj.7042 (PMC6546150; doi:10.7717/peerj.7042)
Supplement: Table S1 [file peerj-07-7042-s001.docx]

|  | Bio1 | Bio2 | Bio3 | Bio4 | Bio5 | Bio6 | Bio7 | Bio8 | Bio9 | Bio10 | Bio11 | Bio12 | Bio13 | Bio14 | Bio15 | Bio16 | Bio17 | Bio18 | Bio19 |
| --- | --- | --- | --- | --- | --- | --- | --- | --- | --- | --- | --- | --- | --- | --- | --- | --- | --- | --- | --- |
| Bio1 | 1 |  |  |  |  |  |  |  |  |  |  |  |  |  |  |  |  |  |  |
| Bio2 | -.130^*^ | 1 |  |  |  |  |  |  |  |  |  |  |  |  |  |  |  |  |  |
| Bio3 | .464^**^ | .379^**^ | 1 |  |  |  |  |  |  |  |  |  |  |  |  |  |  |  |  |
| Bio4 | -.543^**^ | -.142^*^ | **-.958^**^** | 1 |  |  |  |  |  |  |  |  |  |  |  |  |  |  |  |
| Bio5 | .569^**^ | .190^**^ | -.176^**^ | .213^**^ | 1 |  |  |  |  |  |  |  |  |  |  |  |  |  |  |
| Bio6 | **.901^**^** | -.129^*^ | .694^**^ | **-.804^**^** | .276^**^ | 1 |  |  |  |  |  |  |  |  |  |  |  |  |  |
| Bio7 | -.615^**^ | .229^**^ | -.792^**^ | **.923^**^** | .240^**^ | **-.867^**^** | 1 |  |  |  |  |  |  |  |  |  |  |  |  |
| Bio8 | .693^**^ | -.098 | .212^**^ | -.220^**^ | .390^**^ | .482^**^ | -.284^**^ | 1 |  |  |  |  |  |  |  |  |  |  |  |
| Bio9 | .399^**^ | .080 | .331^**^ | -.410^**^ | .290^**^ | .545^**^ | -.400^**^ | -.203^**^ | 1 |  |  |  |  |  |  |  |  |  |  |
| Bio10 | .774^**^ | -.249^**^ | -.170^**^ | .108 | **.848^**^** | .471^**^ | -.035 | .635^**^ | .198^**^ | 1 |  |  |  |  |  |  |  |  |  |
| Bio11 | **.918^**^** | -.007 | .753^**^ | **-.827^**^** | .297^**^ | **.982^**^** | **-.838^**^** | .546^**^ | .489^**^ | .469^**^ | 1 |  |  |  |  |  |  |  |  |
| Bio12 | .251^**^ | -.430^**^ | -.162^**^ | .050 | .118^*^ | .164^**^ | -.105 | .167^**^ | .013 | .310^**^ | .127^*^ | 1 |  |  |  |  |  |  |  |
| Bio13 | .391^**^ | -.016 | .444^**^ | -.501^**^ | .006 | .483^**^ | -.485^**^ | .188^**^ | .261^**^ | .069 | .486^**^ | .684^**^ | 1 |  |  |  |  |  |  |
| Bio14 | .039 | -.573^**^ | -.643^**^ | .556^**^ | .189^**^ | -.236^**^ | .336^**^ | .196^**^ | -.325^**^ | .438^**^ | -.259^**^ | .590^**^ | -.105 | 1 |  |  |  |  |  |
| Bio15 | .301^**^ | .384^**^ | **.806^**^** | -.783^**^ | -.126^*^ | .556^**^ | -.627^**^ | .021 | .424^**^ | -.219^**^ | .581^**^ | -.236^**^ | .497^**^ | **-.842^**^** | 1 |  |  |  |  |
| Bio16 | .365^**^ | -.060 | .395^**^ | -.464^**^ | .001 | .461^**^ | -.465^**^ | .142^*^ | .262^**^ | .068 | .452^**^ | .737^**^ | **.984^**^** | -.069 | .453^**^ | 1 |  |  |  |
| Bio17 | .044 | -.567^**^ | -.652^**^ | .563^**^ | .208^**^ | -.232^**^ | .342^**^ | .198^**^ | -.305^**^ | .450^**^ | -.257^**^ | .619^**^ | -.079 | **.995^**^** | -.843^**^ | -.040 | 1 |  |  |
| Bio18 | .506^**^ | -.204^**^ | .040 | -.081 | .256^**^ | .283^**^ | -.154^**^ | .668^**^ | -.181^**^ | .501^**^ | .344^**^ | .528^**^ | .452^**^ | .468^**^ | -.142^*^ | .414^**^ | .486^**^ | 1 |  |
| Bio19 | -.204^**^ | -.256^**^ | -.293^**^ | .197^**^ | -.037 | -.086 | .068 | -.453^**^ | .279^**^ | -.075 | -.203^**^ | .492^**^ | .293^**^ | .103 | -.041 | .368^**^ | .133^*^ | -.340^**^ | 1 |

Table S1 Correlation analysis of environmental variables

**: significant at the 0.05 level *: significant at the 0.1 level
