# Supplementary figures and images for: Climatic niche comparison across a cryptic species complex
Source: PeerJ. 2019 May 31;7:e7042. doi: 10.7717/peerj.7042 (PMC6546150; doi:10.7717/peerj.7042)

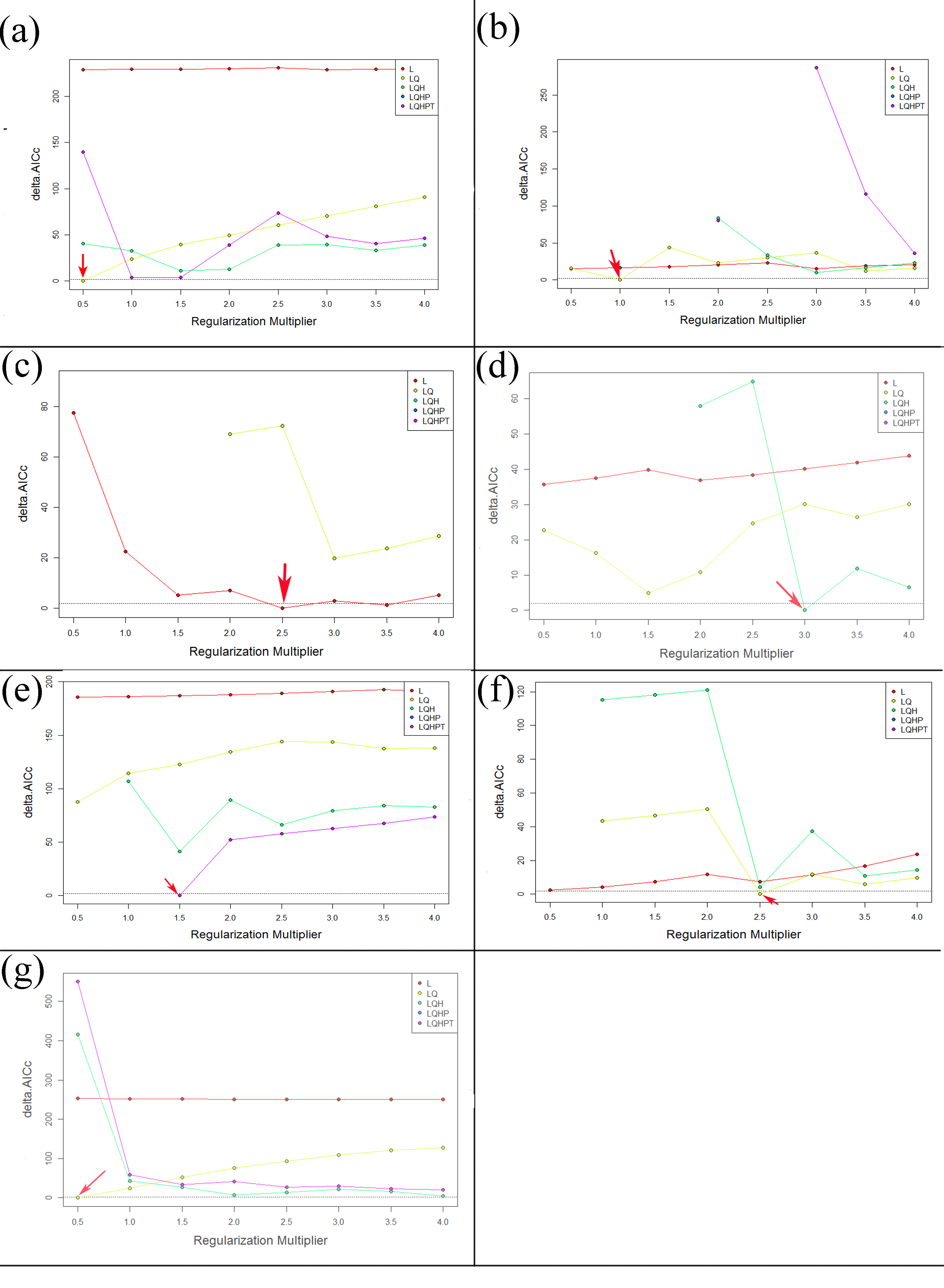

Supplement: Figure S1 — Red arrow indicates the delta AICc-chosen setting. L, linear; Q, Quadratic; H, Hinge; P, Product; T, Threshold [file peerj-07-7042-s003.png]

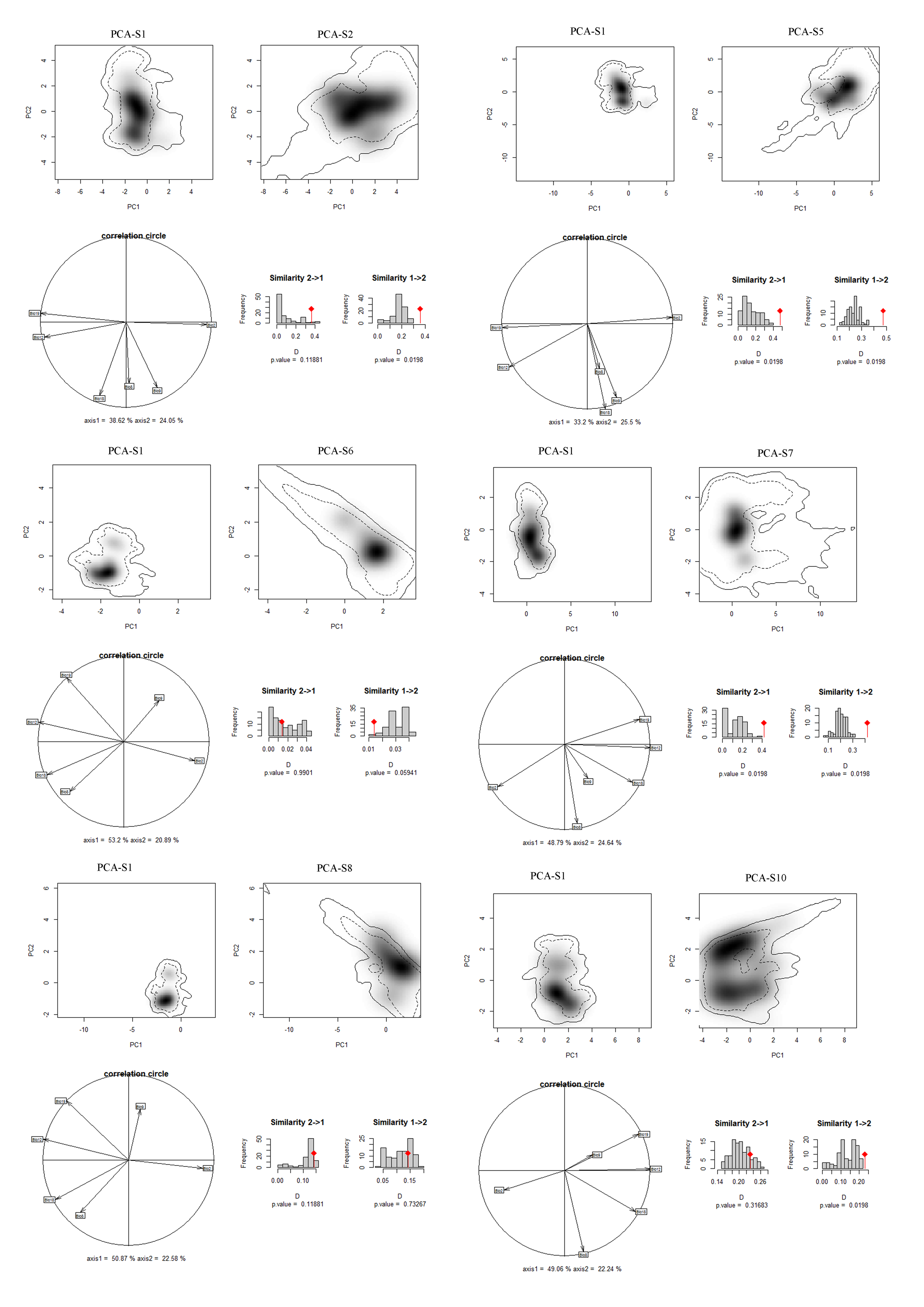

Supplement: Figure S3 — PCA-S1, PCA-S2, PCA-S5, PCA-S6, PCA-S7, PCA-S8, PCA-S10 represent the niche characteristics of the species S1, S2, S5, S6, S7, S8, S10, respectively, along the two-first axes of the PCA, grey shading shows the density of the occurrences of the different species by cell. The solid and dashed contour lines illustrate, respectively, 100% and 50% of the available (background) environment. Panels (correlation circle) represent the contribution of the environmental variables on the first two axes of the PCA and the percentage of the two axes. Histograms show the observed niche overlap (D) between the two species (bars with a diamond) and simulated niche overlaps (grey bars) on which tests of niche similarity are calculated. The significance of the tests is shown by P value. [file peerj-07-7042-s005.png]

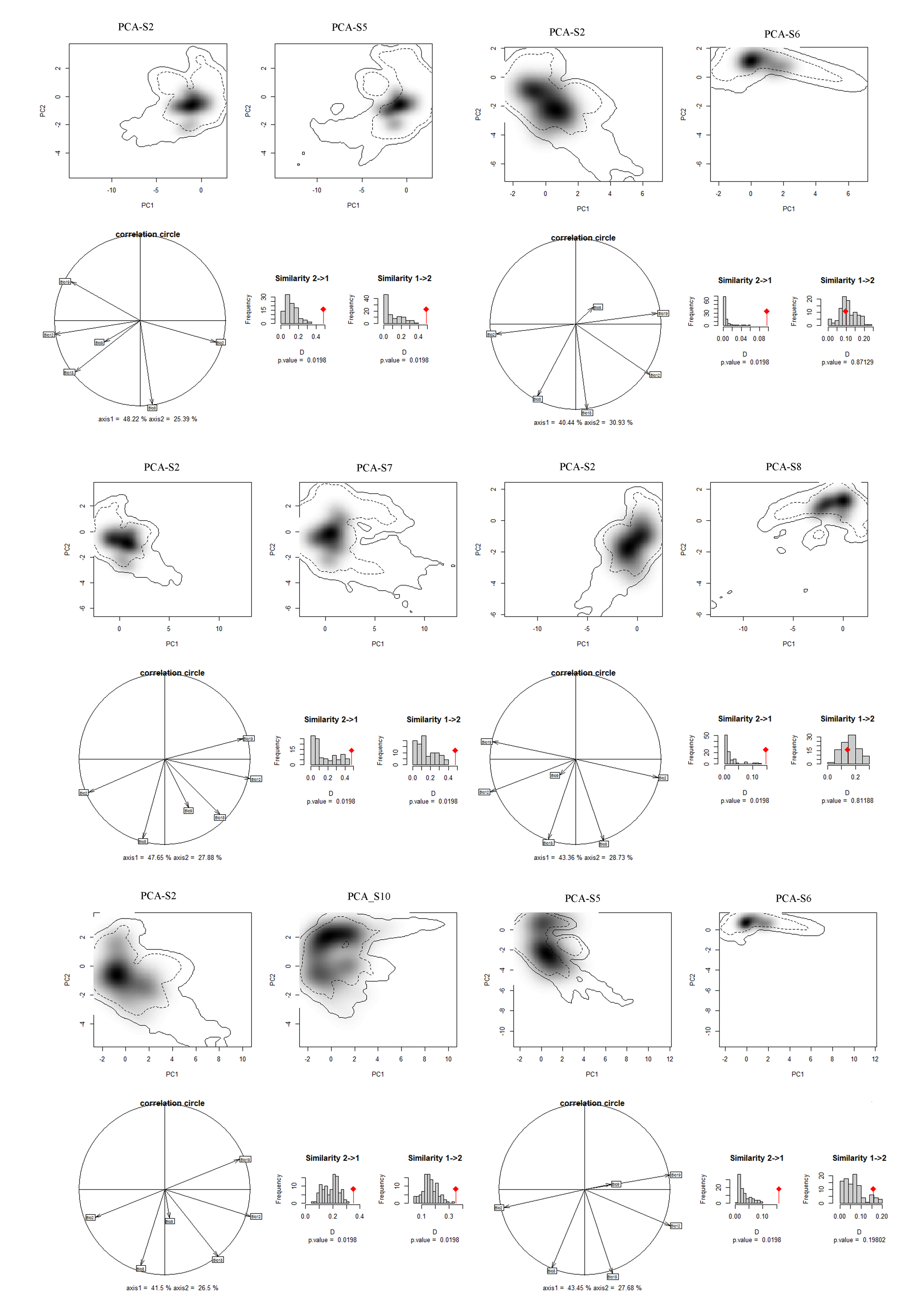

Supplement: Figure S4 — PCA-S2, PCA-S5, PCA-S6, PCA-S7, PCA-S8, PCA-S10 represent the niche characteristics of the species S2, S5, S6, S7, S8, S10, respectively, along the two-first axes of the PCA, grey shading shows the density of the occurrences of the different species by cell. The solid and dashed contour lines illustrate, respectively, 100% and 50% of the available (background) environment. Panels (correlation circle) represent the contribution of the environmental variables on the first two axes of the PCA and the percentage of the two axes. Histograms show the observed niche overlap (D) between the two species (bars with a diamond) and simulated niche overlaps (grey bars) on which tests of niche similarity are calculated. The significance of the tests is shown by P value. [file peerj-07-7042-s006.png]

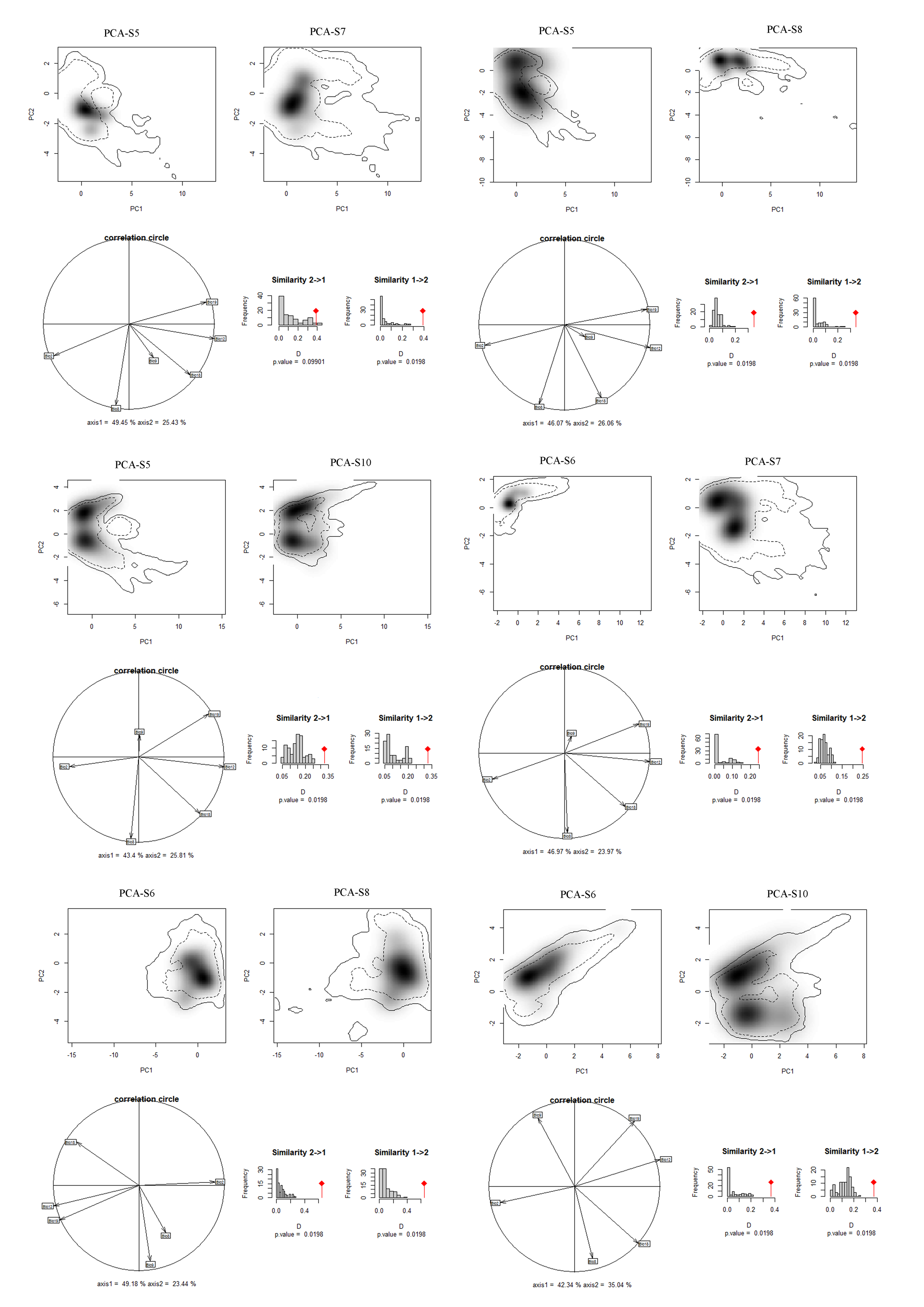

Supplement: Figure S5 — PCA-S5, PCA-S6, PCA-S7, PCA-S8, PCA-S10 represent the niche characteristics of the species S5, S6, S7, S8, S10, respectively, along the two-first axes of the PCA, grey shading shows the density of the occurrences of the different species by cell. The solid and dashed contour lines illustrate, respectively, 100% and 50% of the available (background) environment. Panels (correlation circle) represent the contribution of the environmental variables on the first two axes of the PCA and the percentage of the two axes. Histograms show the observed niche overlap (D) between the two species (bars with a diamond) and simulated niche overlaps (grey bars) on which tests of niche similarity are calculated. The significance of the tests is shown by P value. [file peerj-07-7042-s007.png]

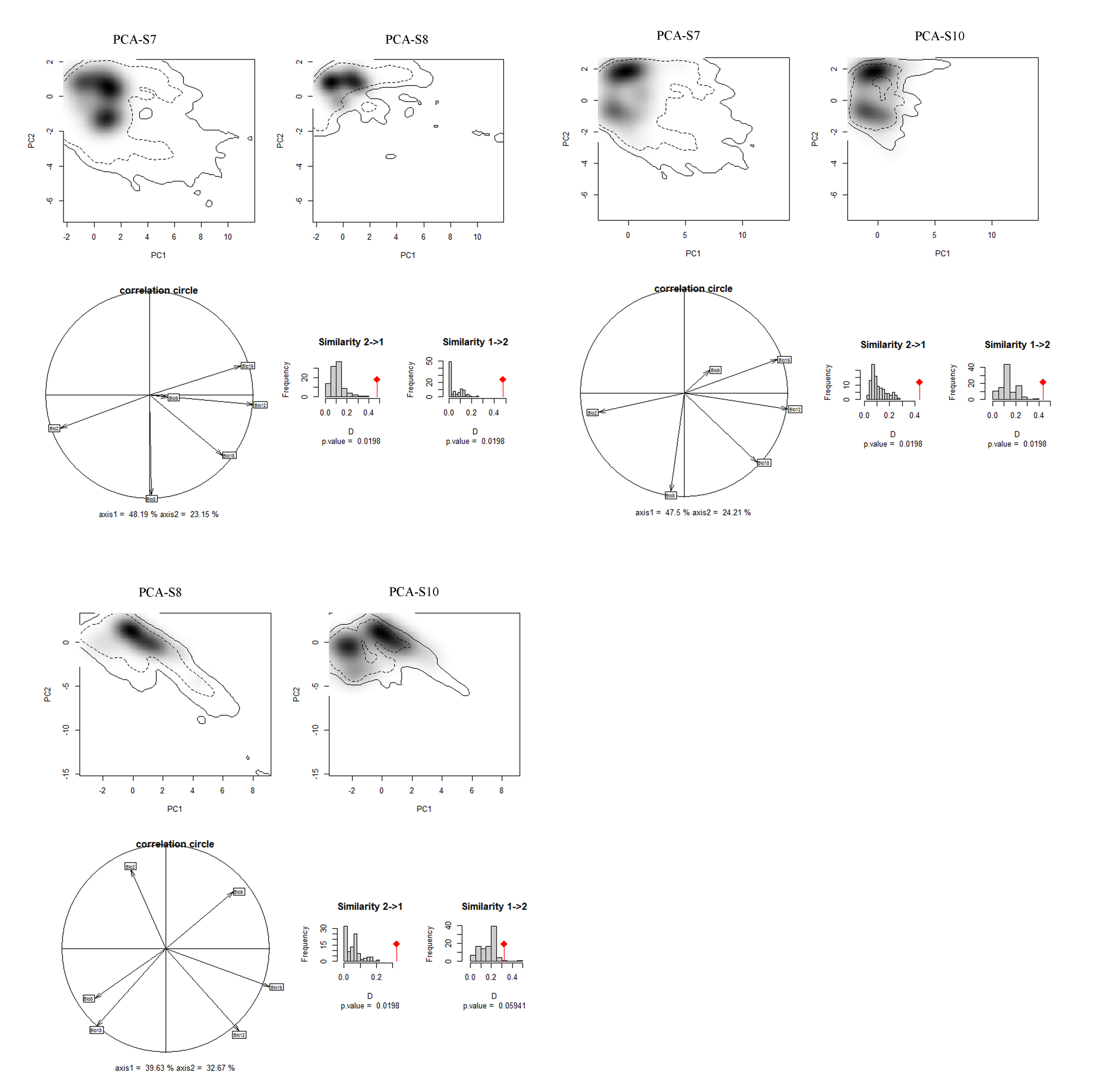

Supplement: Figure S6 — PCA-S7, PCA-S8, PCA-S10 represent the niche characteristics of the species S5, S6, S7, S8, S10, respectively, along the two-first axes of the PCA, grey shading shows the density of the occurrences of the different species by cell. The solid and dashed contour lines illustrate, respectively, 100% and 50% of the available (background) environment. Panels (correlation circle) represent the contribution of the environmental variables on the first two axes of the PCA and the percentage of the two axes. Histograms show the observed niche overlap (D) between the two species (bars with a diamond) and simulated niche overlaps (grey bars) on which tests of niche similarity are calculated. The significance of the tests is shown by P value. [file peerj-07-7042-s008.png]

**Figure S7. Result of identify test by ENMtools**

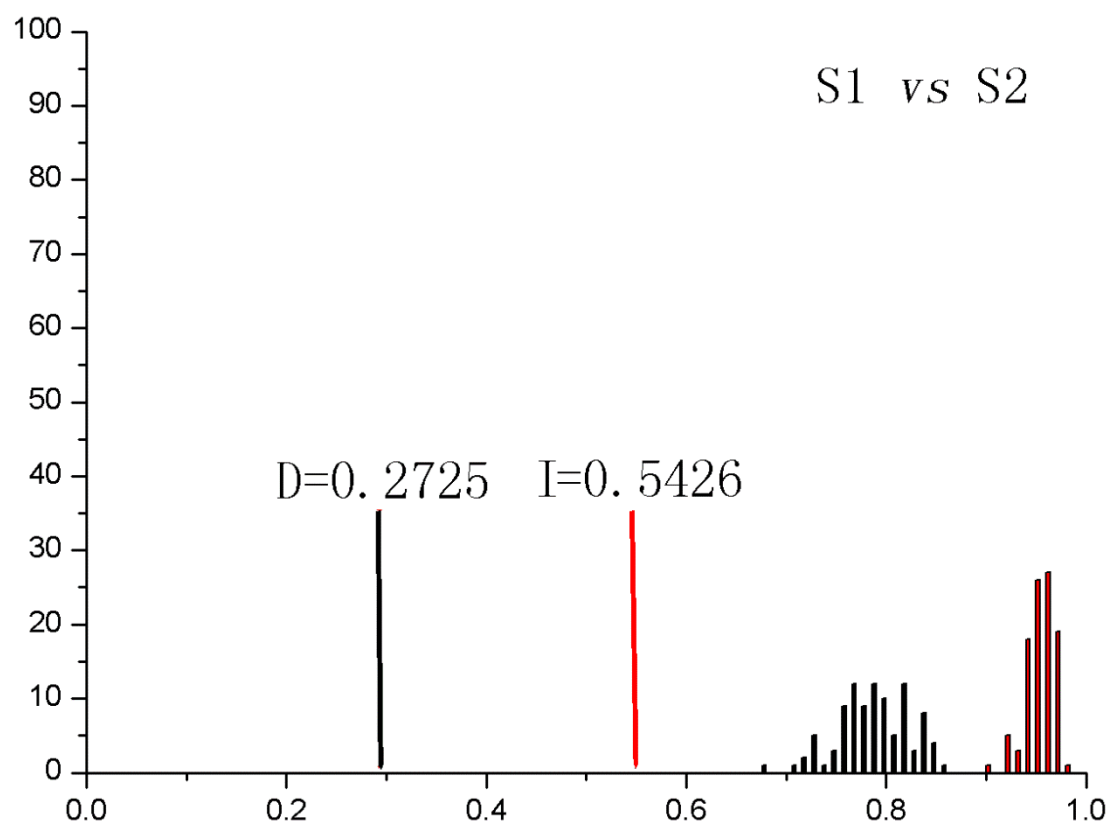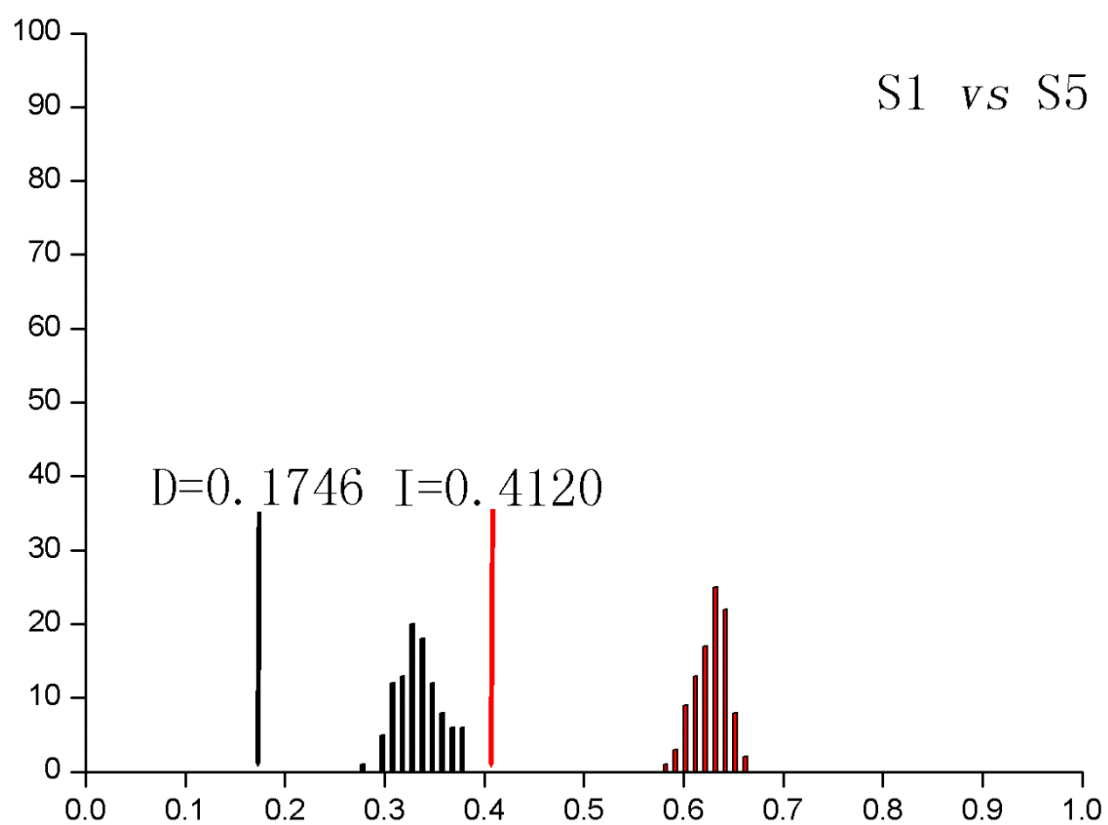

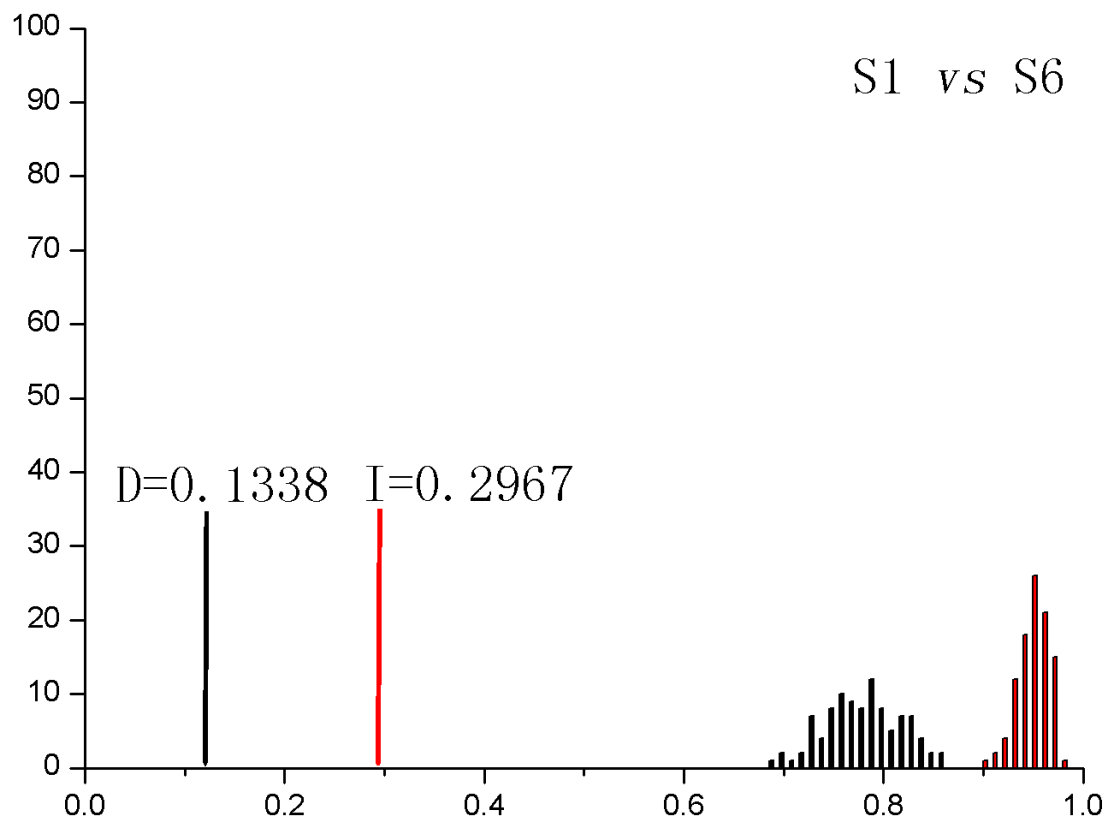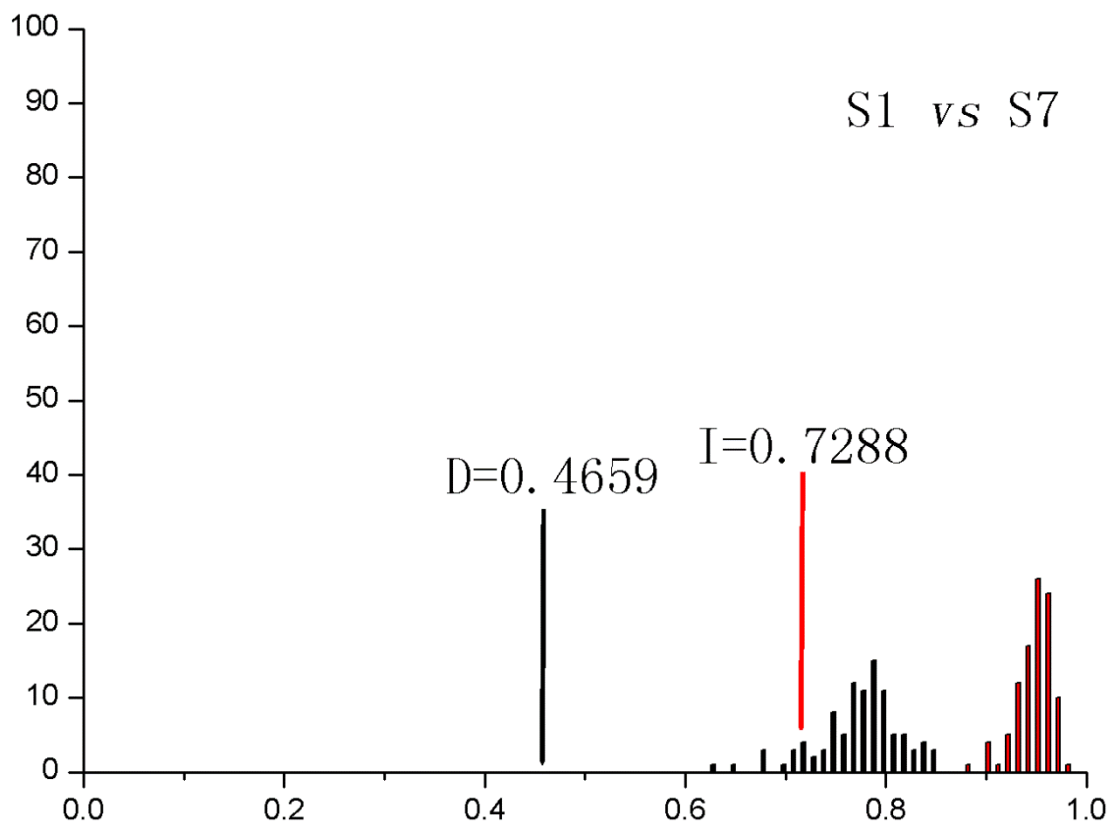

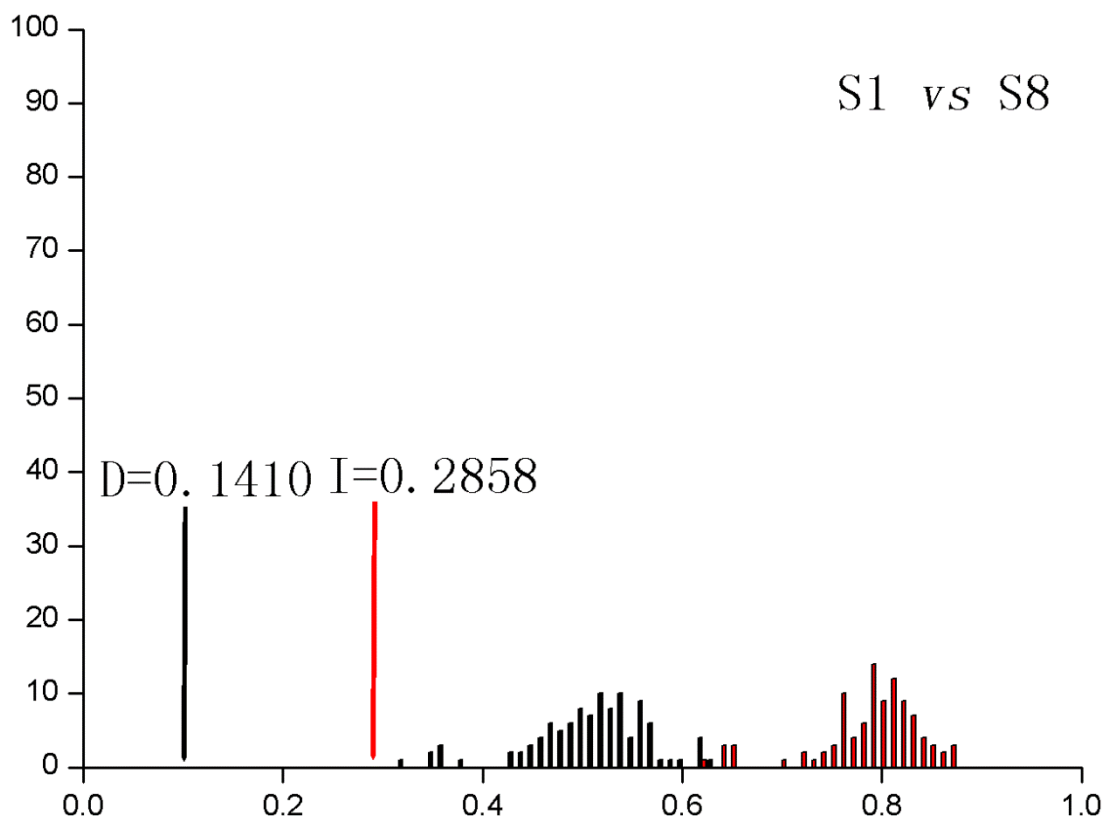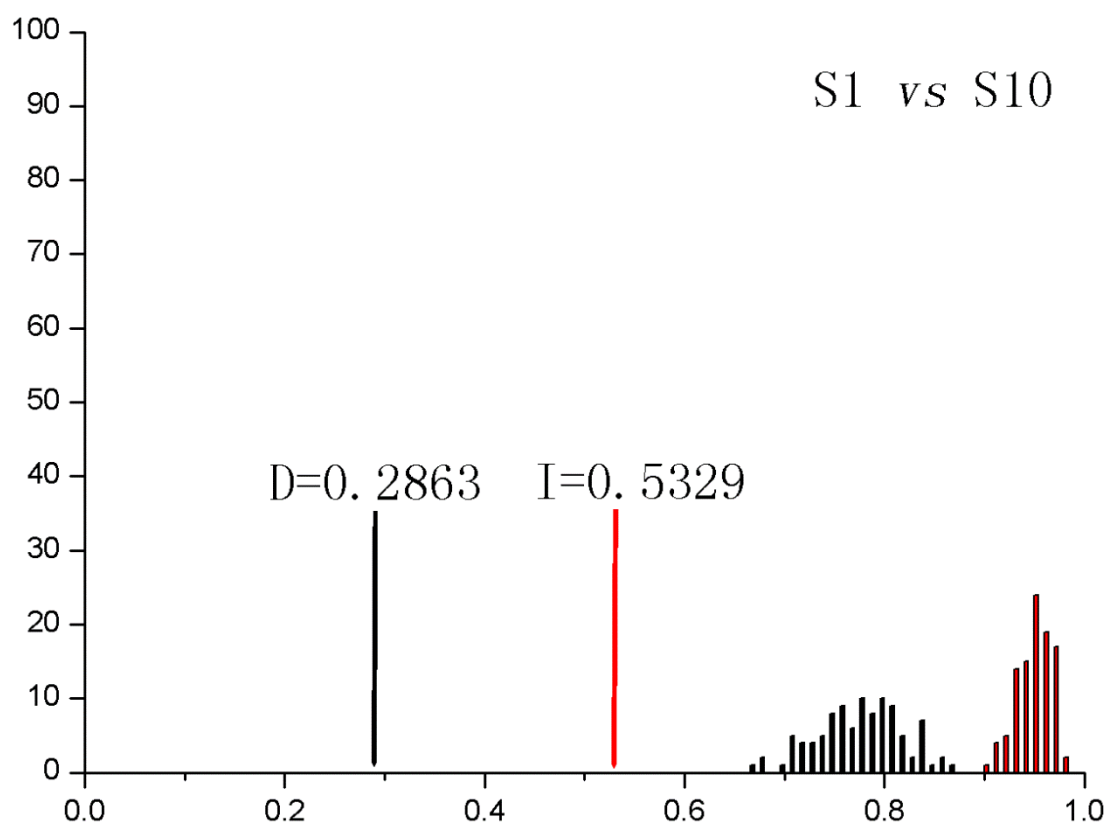

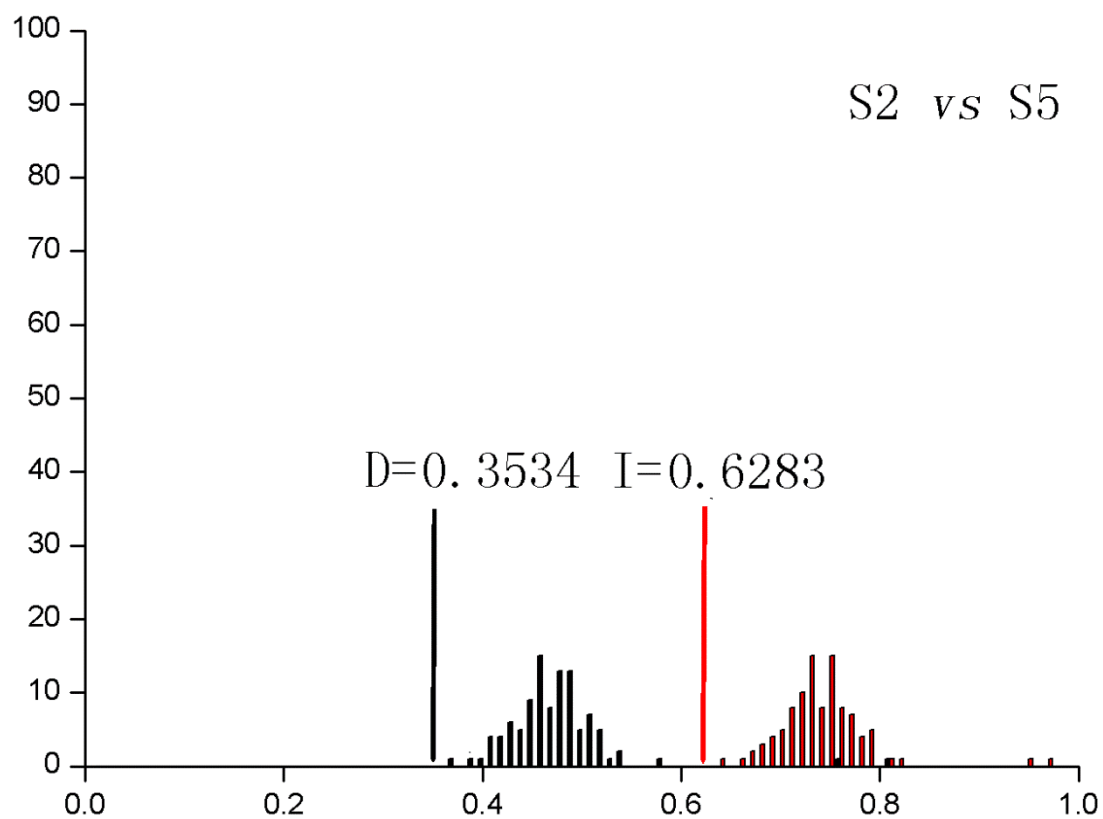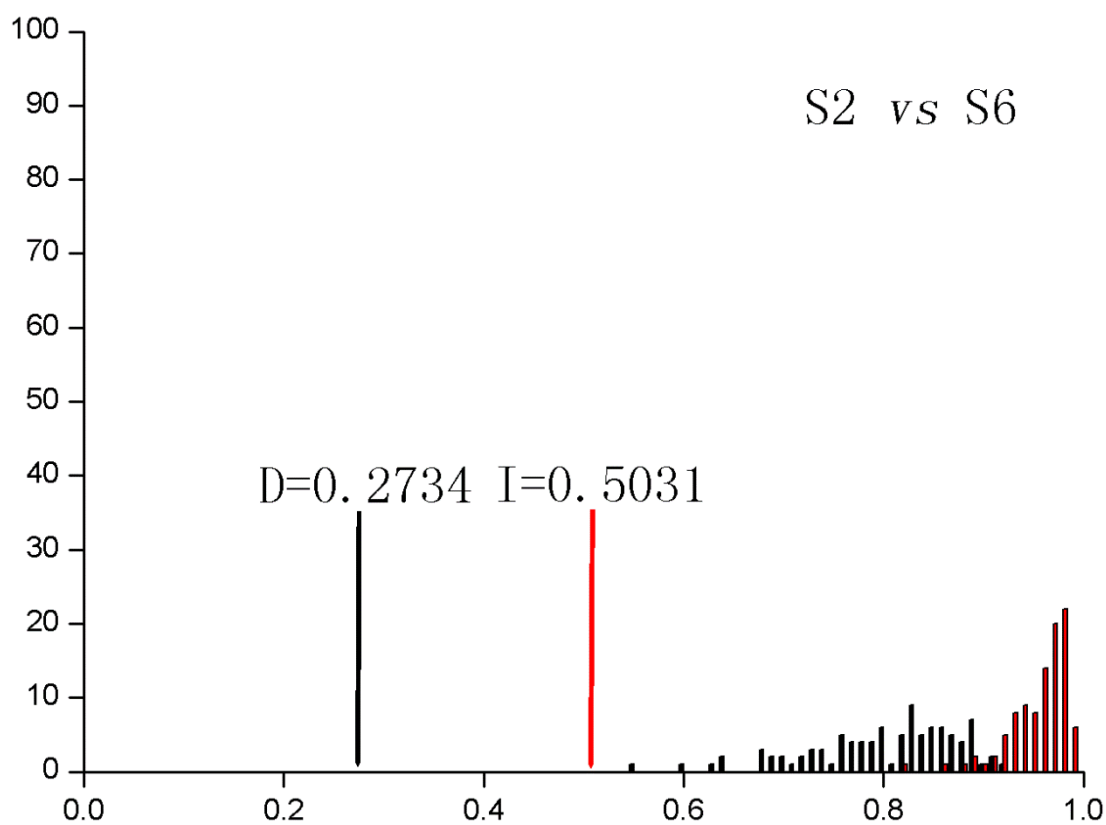

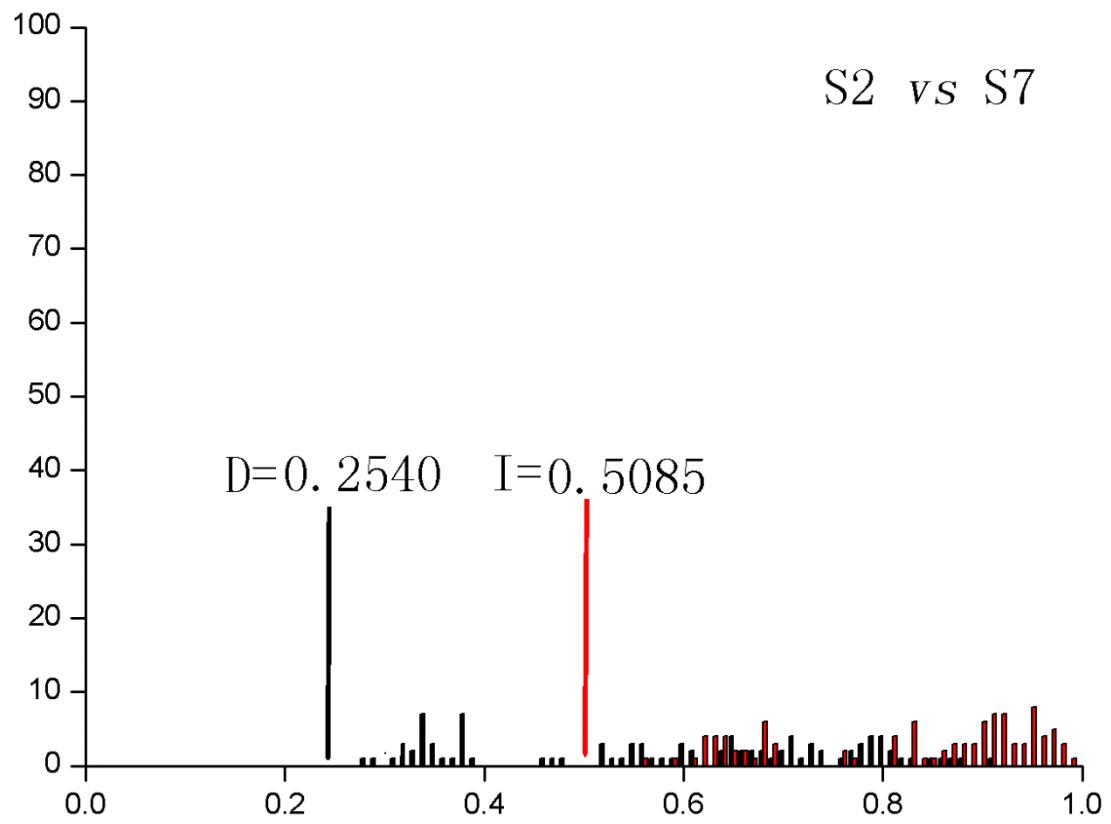

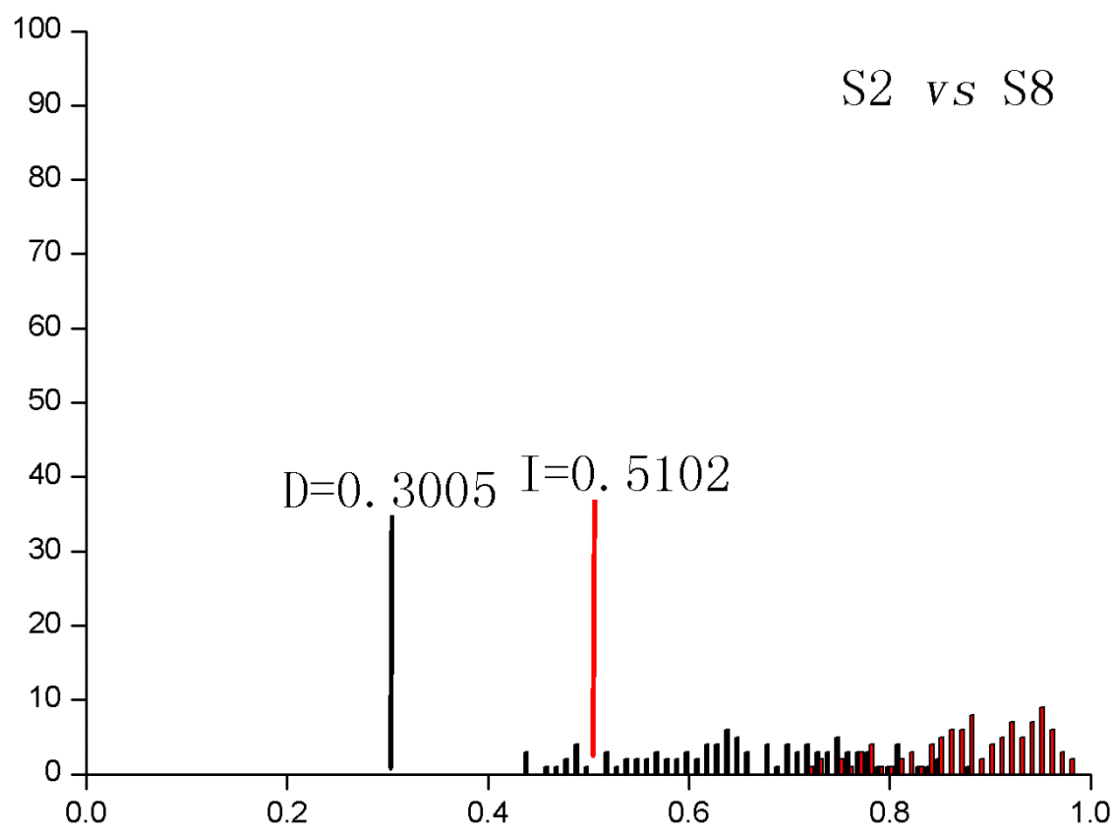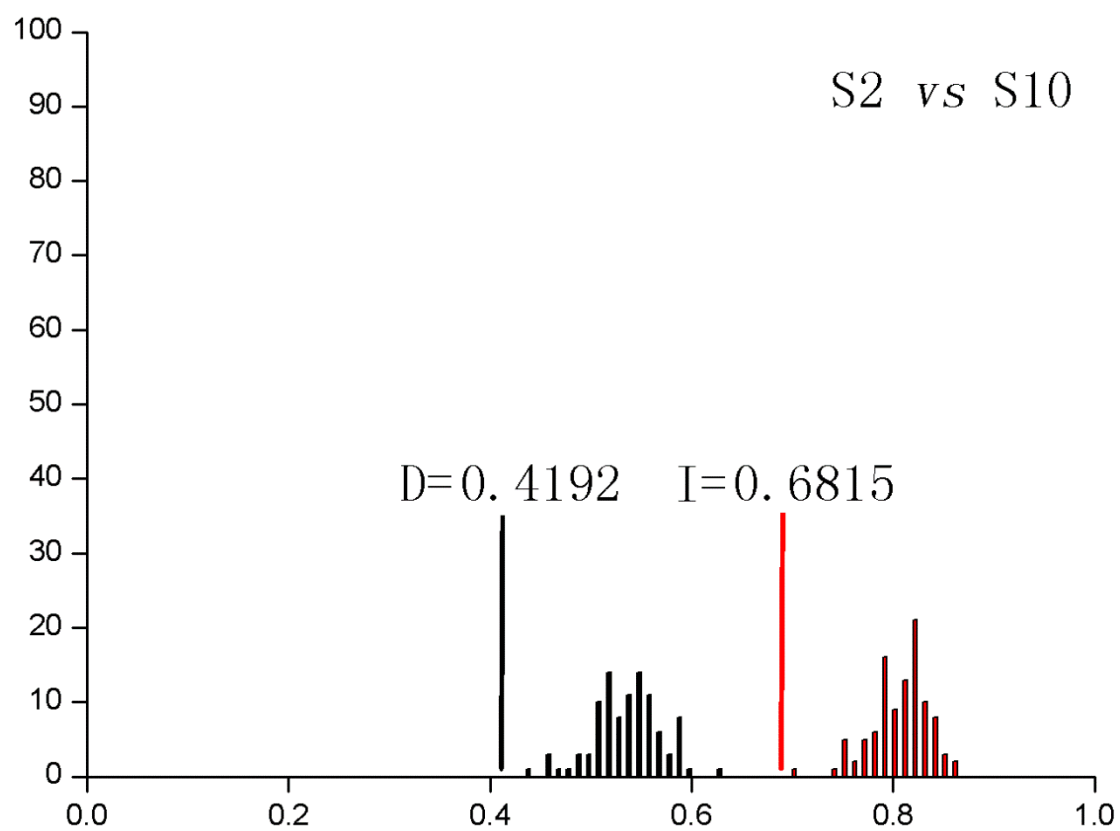

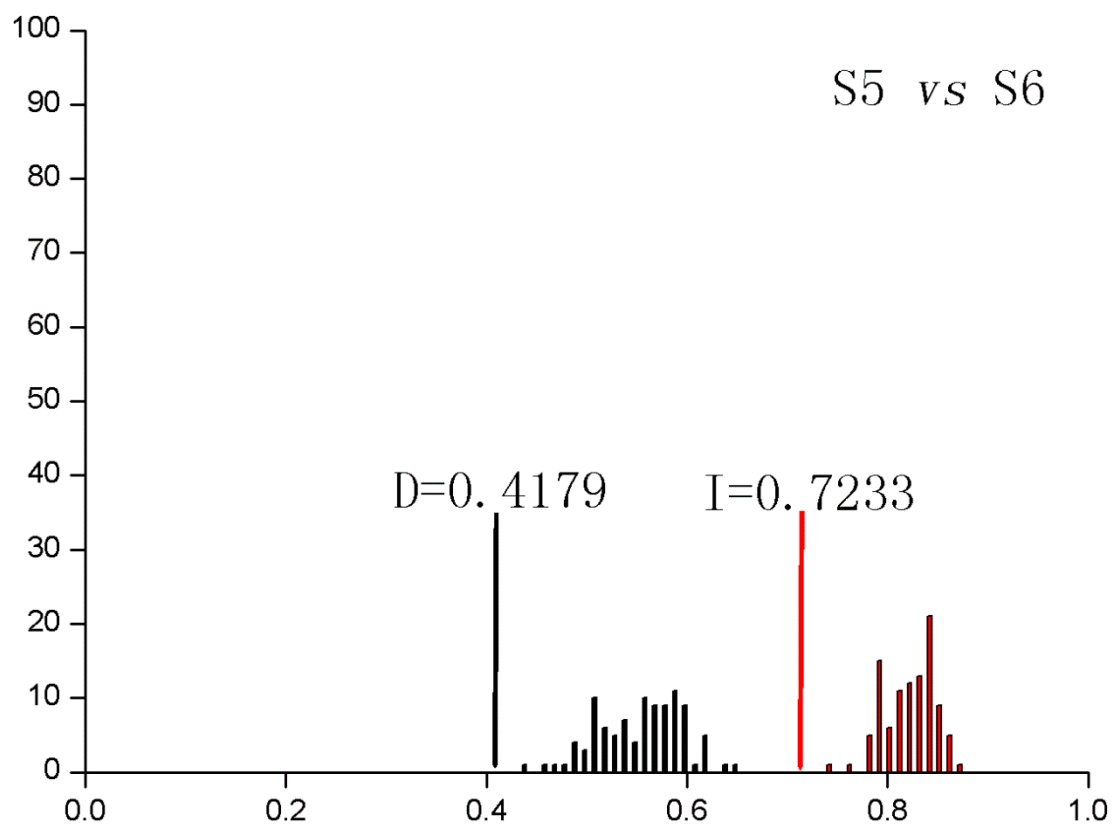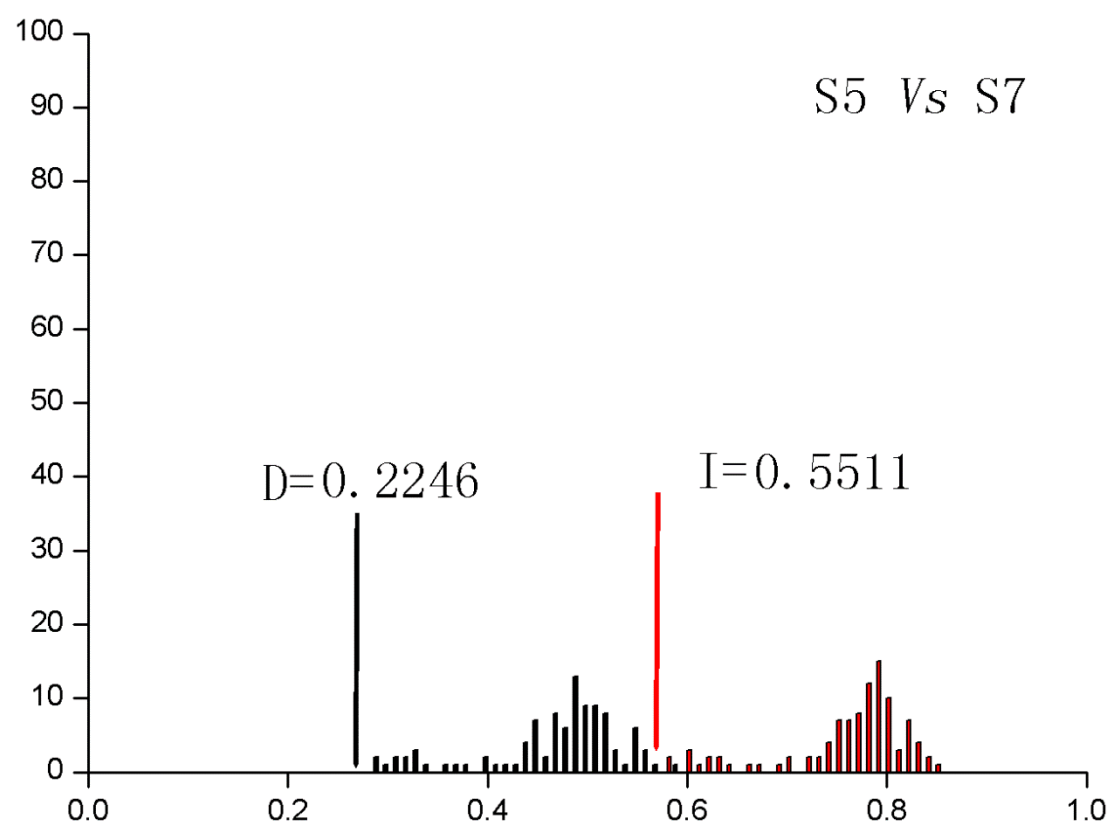

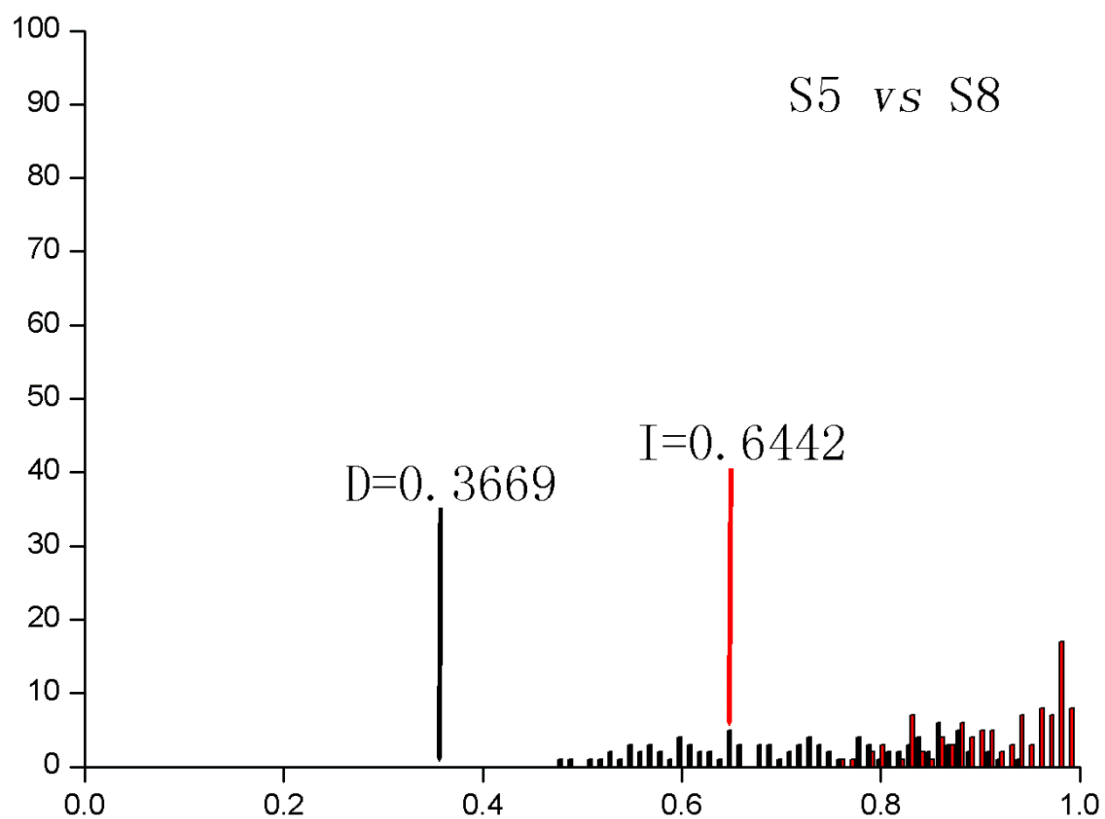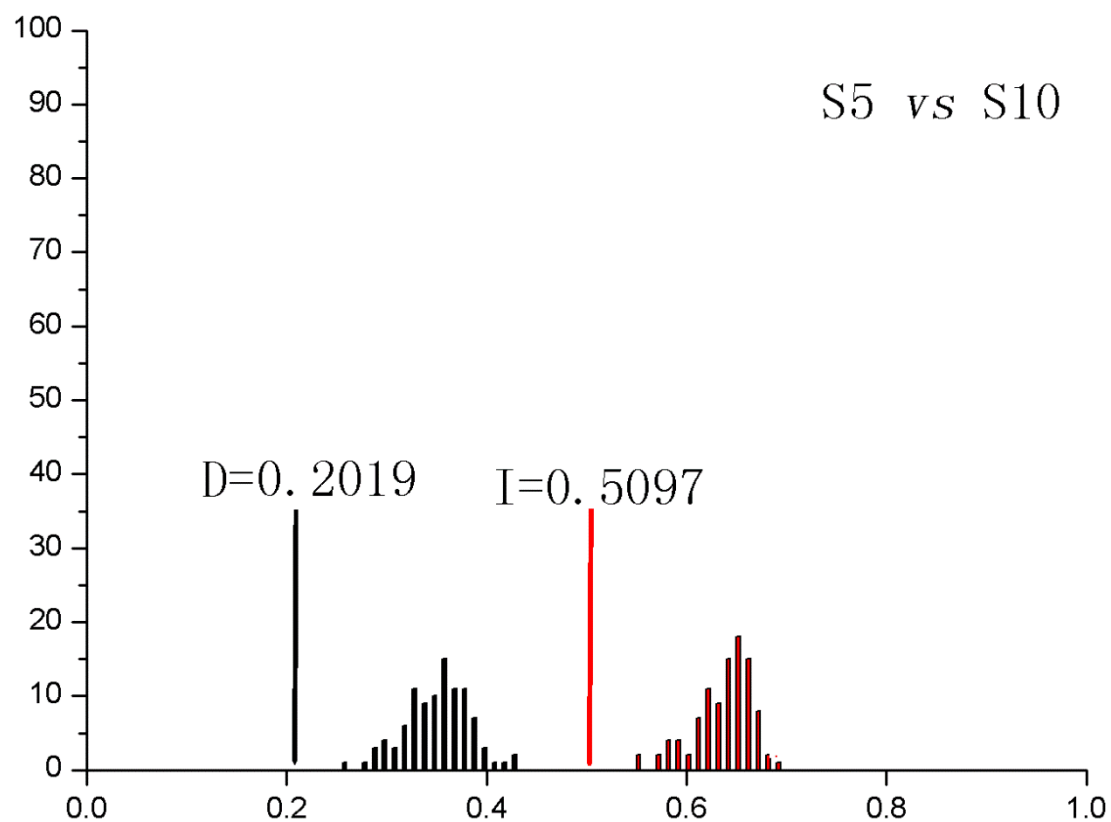

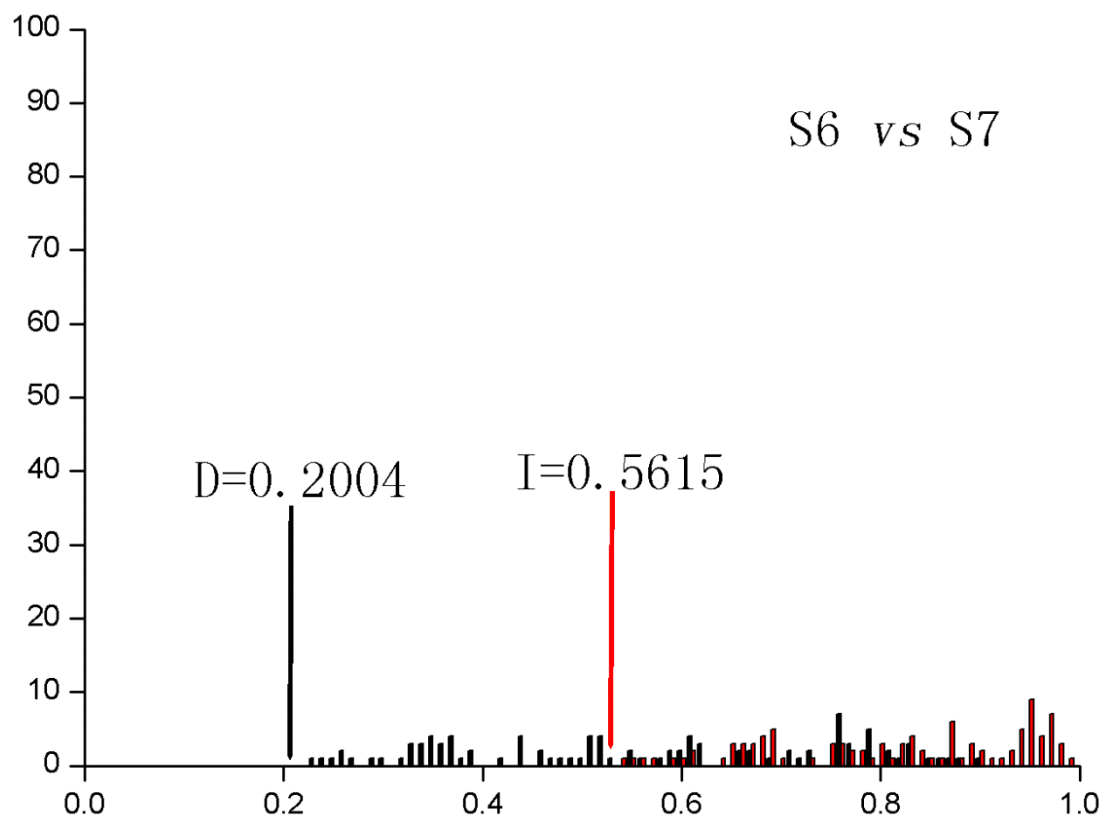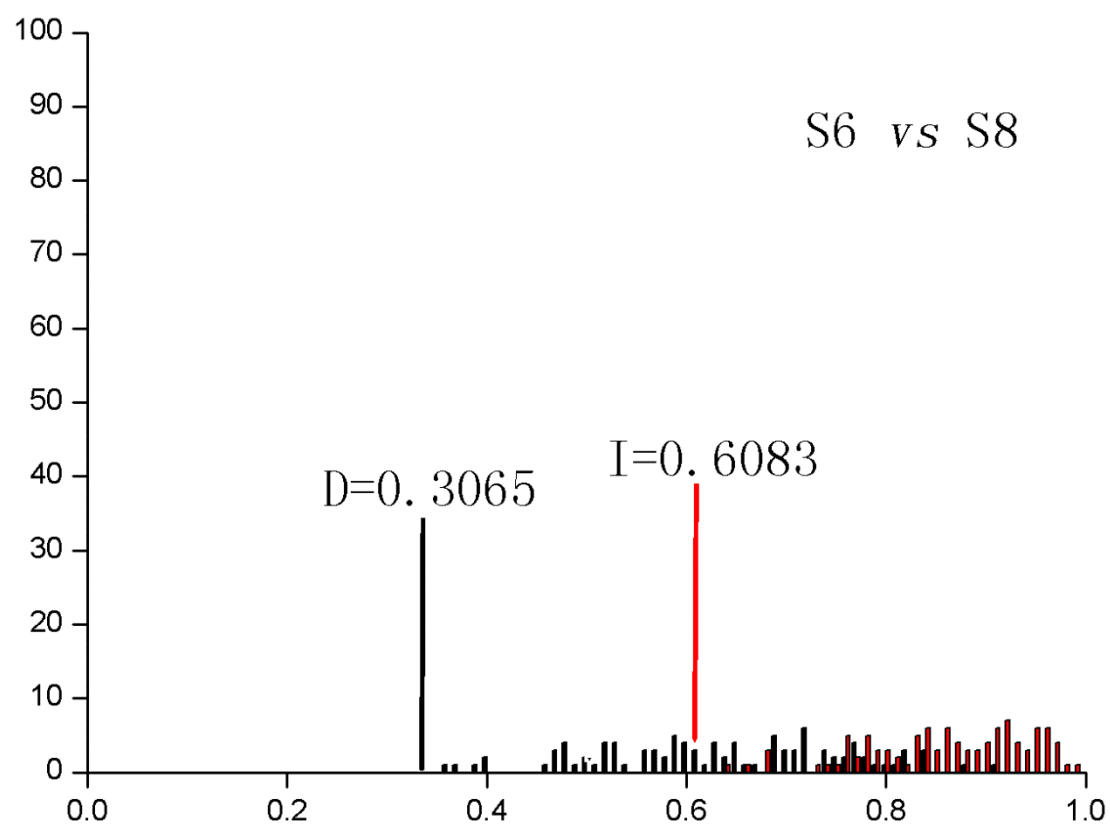

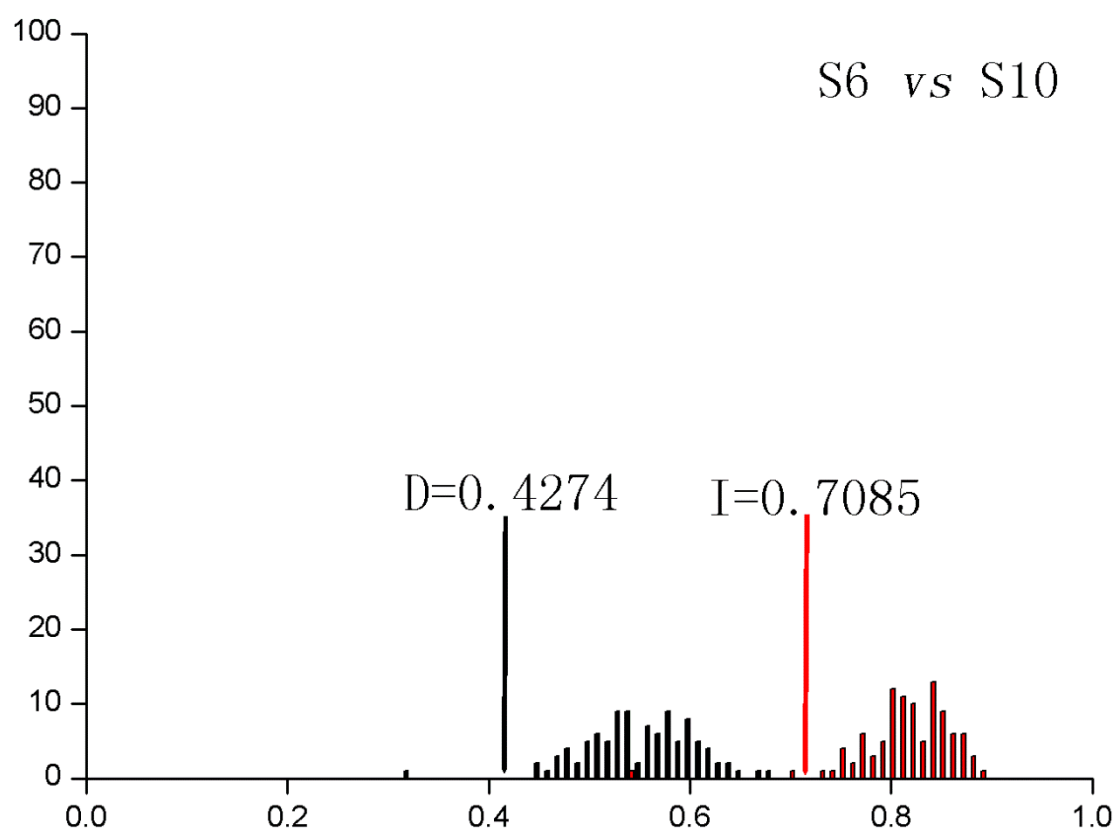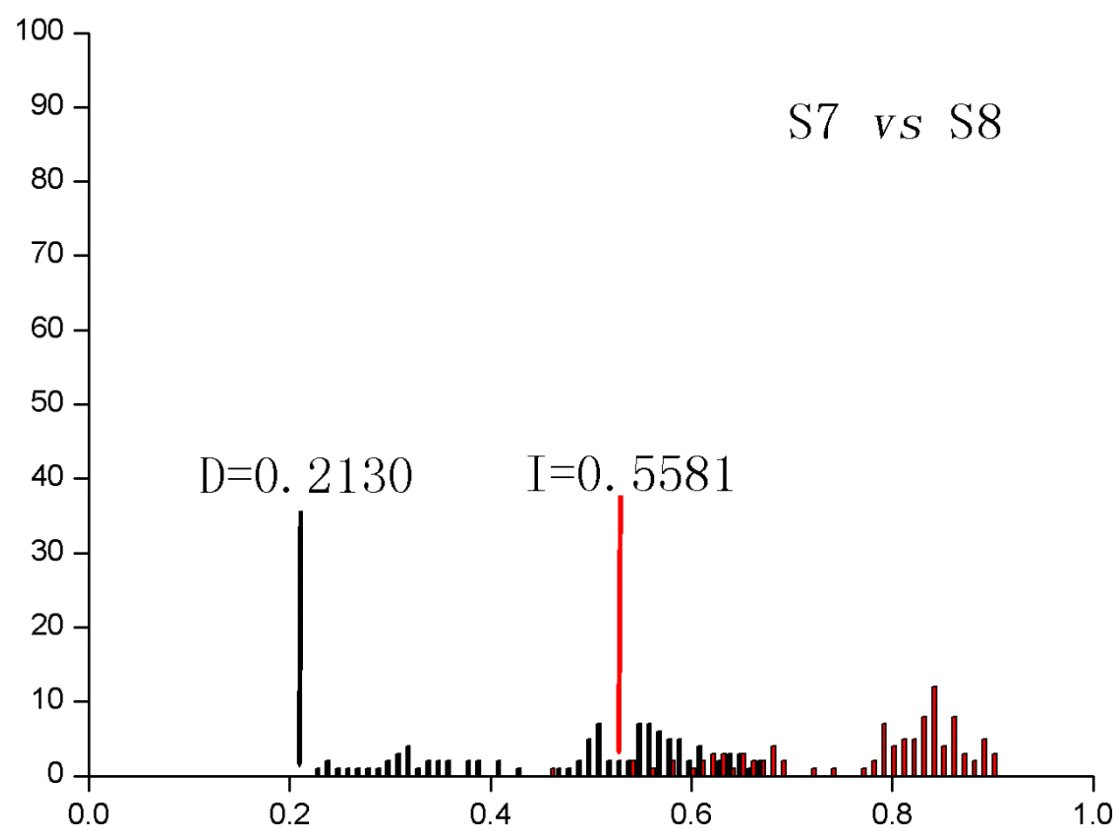

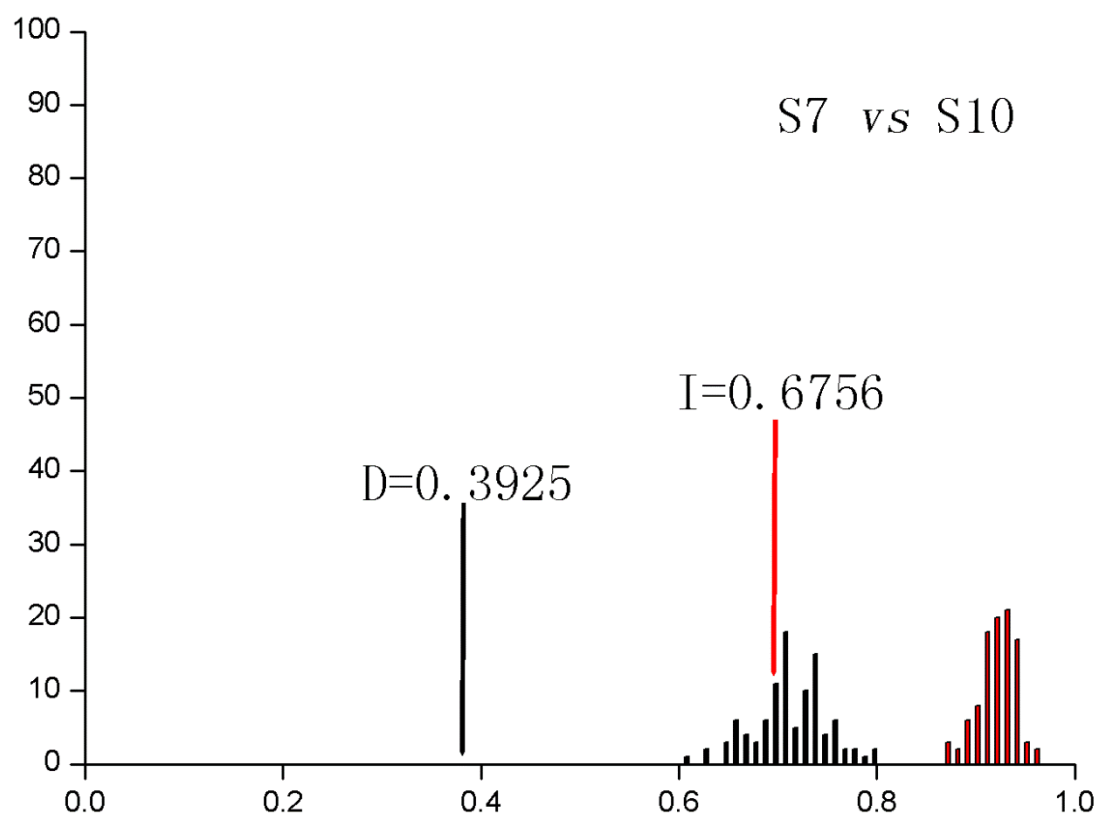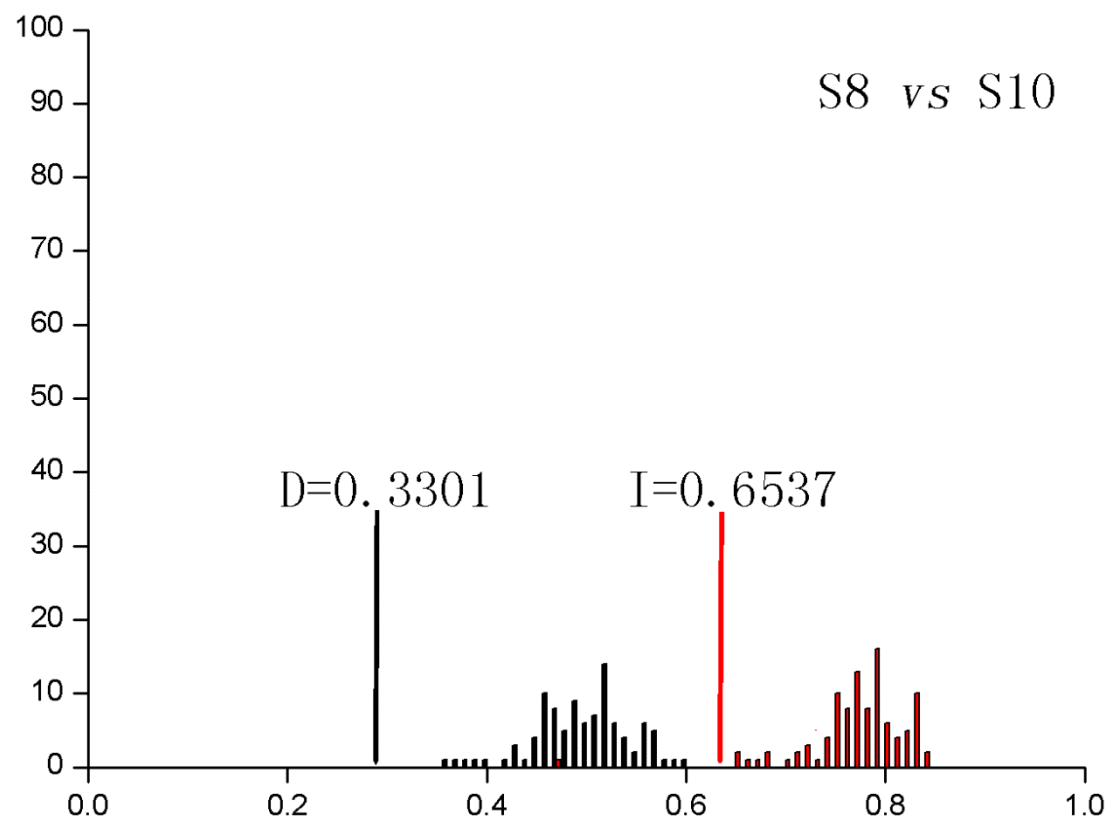

Supplement: Figure S7 [file peerj-07-7042-s009.pdf]
