# Supplementary material for: Climatic niche comparison across a cryptic species complex
Source: PeerJ. 2019 May 31;7:e7042. doi: 10.7717/peerj.7042 (PMC6546150; doi:10.7717/peerj.7042)

Figure S2. Partial-ROC values and graphics; null model (red distribution), distribution of expectations created via bootstrapping replacement of 50% of the total available points and 1,000 resampling replicates (blue distribution).

S1: Mean AUC ratio after 1000 simulations: 1.986836 (P.value=0)

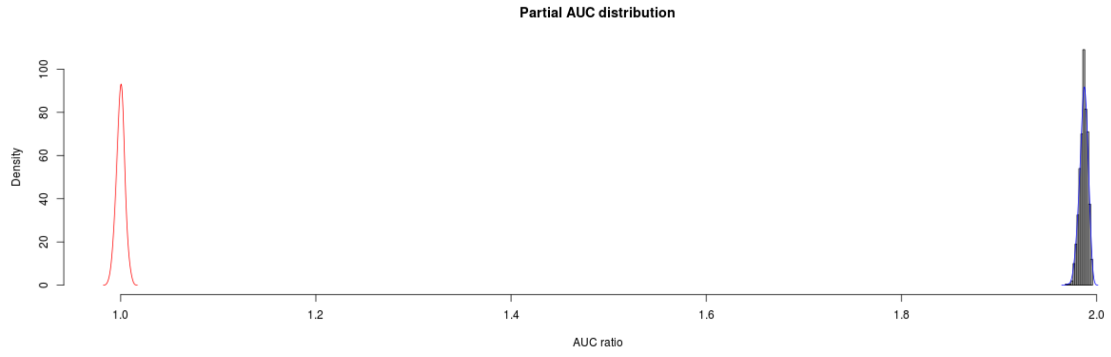

S2: Mean AUC ratio after 1000 simulations: 1.984517 (P.value=0)

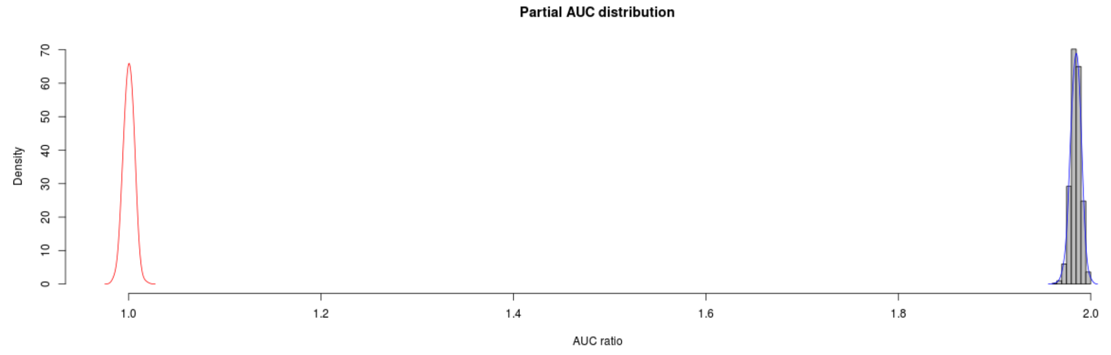

S5: Mean AUC ratio after 1000 simulations: 1.929322 (P.value=0)

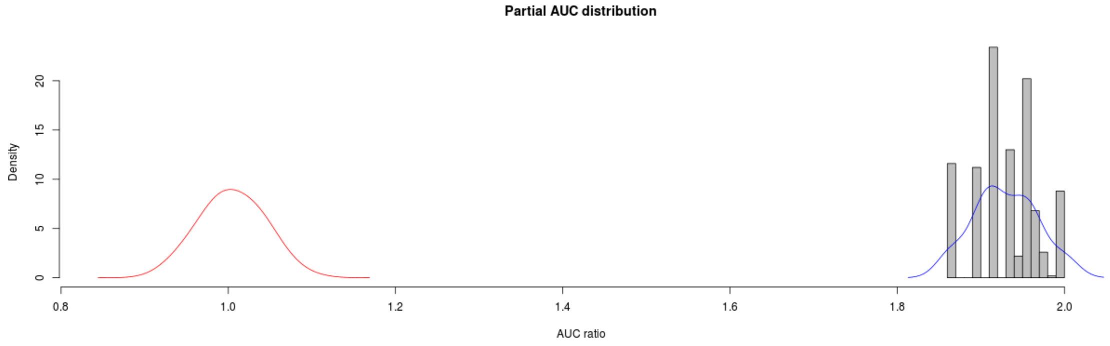

S6: Mean AUC ratio after 1000 simulations: 1.97818 （P.value=0）

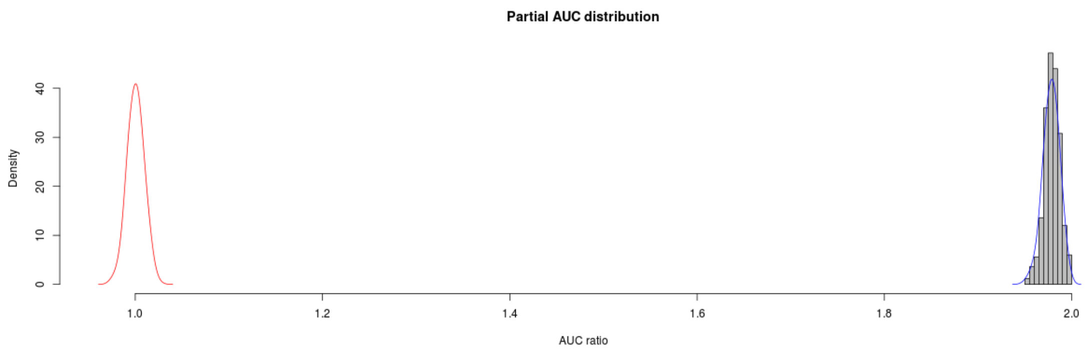

S7: Mean AUC ratio after 1000 simulations: 1.976526 （P.value=0）

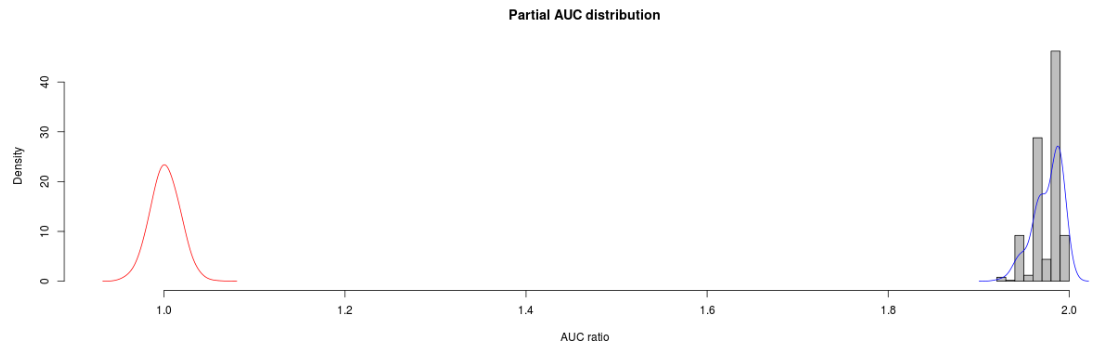

S8: Mean AUC ratio after 1000 simulations: 1.991804 （P.value=0）

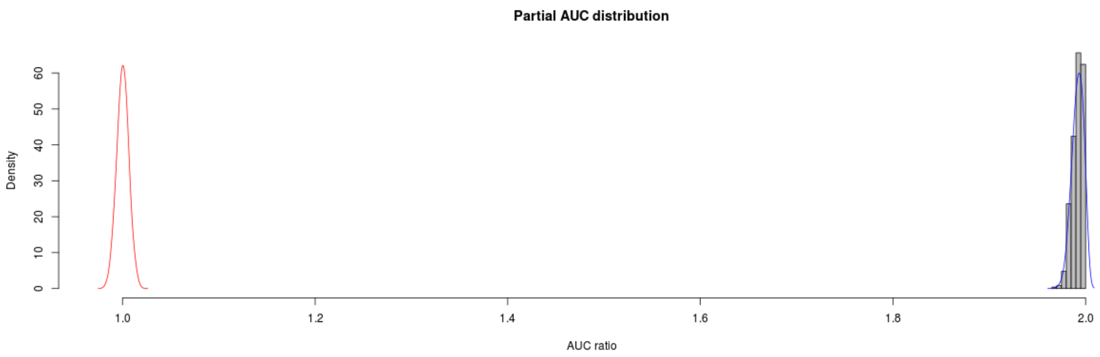

S10: Mean AUC ratio after 1000 simulations: 1.93884 （P.value=0）

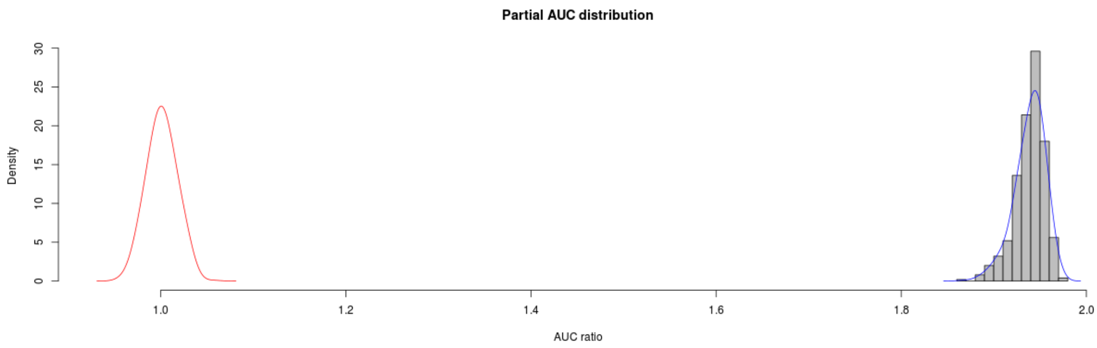

Supplement: Figure S2 — Partial-ROC values and graphics; null model (red distribution), distribution of expectations created via bootstrapping replacement of 50% of the total available points and 1,000 resampling replicates (blue distribution). [file peerj-07-7042-s004.pdf]
